# Vangl2, a planar cell polarity molecule, is implicated in irreversible and reversible kidney glomerular injury

**DOI:** 10.1002/path.5158

**Published:** 2018-11-16

**Authors:** Eugenia Papakrivopoulou, Elisavet Vasilopoulou, Maja T Lindenmeyer, Sabrina Pacheco, Hortensja Ł Brzóska, Karen L Price, Maria Kolatsi‐Joannou, Kathryn E White, Deborah J Henderson, Charlotte H Dean, Clemens D Cohen, Alan D Salama, Adrian S Woolf, David A Long

**Affiliations:** ^1^ Developmental Biology and Cancer Programme UCL Great Ormond Street Institute of Child Health London UK; ^2^ Medway School of Pharmacy University of Kent Chatham Maritime UK; ^3^ Nephrological Center, Medical Clinic and Policlinic IV University of Munich Munich Germany; ^4^ Department of Medicine University Medical Center Hamburg‐Eppendorf Hamburg Germany; ^5^ Electron Microscopy Research Services Newcastle University Newcastle upon Tyne UK; ^6^ Cardiovascular Research Centre Institute of Genetic Medicine, Newcastle University Newcastle upon Tyne UK; ^7^ Inflammation Repair and Development Section National Heart and Lung Institute, Imperial College London London UK; ^8^ University College London Centre for Nephrology, Royal Free Hospital London UK; ^9^ Faculty of Biology Medicine and Health School of Biological Sciences, University of Manchester Manchester UK; ^10^ Royal Manchester Children's Hospital, Manchester University NHS Foundation Trust, Manchester Academic Health Science Centre Manchester UK

**Keywords:** glomerulus, kidney disease, matrix metalloproteinase, planar cell polarity, podocyte

## Abstract

Planar cell polarity (PCP) pathways control the orientation and alignment of epithelial cells within tissues. Van Gogh‐like 2 (Vangl2) is a key PCP protein that is required for the normal differentiation of kidney glomeruli and tubules. Vangl2 has also been implicated in modifying the course of acquired glomerular disease, and here, we further explored how Vangl2 impacts on glomerular pathobiology in this context. Targeted genetic deletion of *Vangl2* in mouse glomerular epithelial podocytes enhanced the severity of not only irreversible accelerated nephrotoxic nephritis but also lipopolysaccharide‐induced reversible glomerular damage. In each proteinuric model, genetic deletion of *Vangl2* in podocytes was associated with an increased ratio of active‐MMP9 to inactive MMP9, an enzyme involved in tissue remodelling. In addition, by interrogating microarray data from two cohorts of renal patients, we report increased *VANGL2* transcript levels in the glomeruli of individuals with focal segmental glomerulosclerosis, suggesting that the molecule may also be involved in certain human glomerular diseases. These observations support the conclusion that Vangl2 modulates glomerular injury, at least in part by acting as a brake on MMP9, a potentially harmful endogenous enzyme. © 2018 The Authors. *The Journal of Pathology* published by John Wiley & Sons Ltd on behalf of Pathological Society of Great Britain and Ireland.

## Introduction

*Van Gogh‐like 2* (Vangl2) regulates planar cell polarity (PCP), controlling orientation and alignment of epithelial cells within tissues 1. PCP is implicated in heart 2, lung 3, neural tube 4, 5 and blood vessel 6 development. In kidneys, Vangl2 is expressed in epithelial podocytes in forming glomeruli, the blood ultrafiltration units, and in nephron and collecting duct tubules 7, 8. Homozygous *Loop‐tail* mice with *Vangl2* ^*Lp*^ point mutations have malformed kidneys with a paucity of collecting ducts and dysmorphic glomeruli 9, 10. Although *Vangl2* ^*Lp/+*^ kidneys develop normally, compound heterozygotes harbouring *Vangl2* ^*Lp*^ and a point mutation in another PCP gene, *Cadherin EGF LAG seven‐pass G‐type receptor 1* (*Celsr1*), have branching malformations 11.

PCP is also implicated in acquired kidney disease. Mitotic orientation, a PCP‐mediated process, is aberrant in kidney cystogenesis 12. Glomerular *Vangl2* transcripts increased 48 h after the initiation of kidney injury by nephrotoxic nephritis (NTN), a progressive disease model, and NTN is more severe in mice with podocyte‐specific *Vangl2* deletion 8. However, how *Vangl2* modulates glomerular injury is unclear. Possibly, *Vangl2* attenuates NTN‐induced podocyte depletion 8. Alternatively, *Vangl2* might modulate tissue remodelling. Indeed, Vangl2 alters the activity of matrix metalloproteinases (MMPs). *Vangl2* downregulation in zebrafish causes increased MMP14 availability, with reduced extracellular matrix (ECM) and disrupted convergent extension 13. *Vangl2* ^*Lp/+*^ mice also have both increased *Mmp12* transcripts and active protein levels in their lungs 14. Moreover, glomerular podocytes express MMP2 and MMP9 15, 16; the latter is upregulated in NTN 17, and experimentally downregulating MMP9 modulates NTN 17, 18.

We hypothesised that Vangl2 impacts on glomerular disease by modulating MMP. We tested this by analysing mouse models of irreversible and reversible glomerular injury, both accompanied by leakage of protein into the urine. Irreversible injury was examined in NTN mice, analogous to humans with focal segmental glomerulosclerosis (FSGS). Injection of lipopolysaccharide (LPS) in mice was used to induce reversible glomerular injury, as occurs in humans with minimal change disease (MCD). Our results support the conclusion that Vangl2 modulates glomerular injury, in part by acting as a brake on MMP9.

## Methods

### Transgenic mice

All procedures were approved by the UK Home Office. For specific gene deletion in glomerular podocytes, we used *PodCre* mice that express *Cre* recombinase driven by the promoter of *podocin*, a gene expressed in podocytes from the immature capillary loop stage of glomerular development to maturity 19. Initially, we examined the specificity of *Cre* recombination by breeding *PodCre* ^*+*^ mice with *R26R‐EYFP* mice, which have a *loxP*‐flanked STOP sequence followed by the enhanced yellow fluorescent protein gene (EYFP) inserted into the *Gt(ROSA)26Sor* locus. Subsequently, to delete *Vangl2* in podocytes, we crossed *PodCre* ^*+*^ mice with *Vangl2* ^*flox/flox*^ mice 20, where *loxP* sites flank exon 4, with *PodCre* ^*+*^ */Vangl2* ^*/flox/+*^ mice being mated to generate *PodCre* ^*+*^ */Vangl2* ^*flox/flox*^ mice and littermate controls, *Vangl2* ^*flox/flox*^ without *Cre*. Primers to detect the *PodCre*, *Vangl2* ^*flox*^ and excised exon 4 of *Vangl2* (Δ band) alleles are detailed in the supplementary material, Supplementary materials and methods. Recombination of *Vangl2* by *Cre* generates a premature stop codon that gives rise to a protein lacking the four trans‐membrane domains and the C‐terminal PDZ‐binding domain required for the interaction of Vangl2 with other proteins 21, 22. All transgenic mouse strains were on a C57Bl/6 background for >10 generations.

### Murine models of glomerular disease

To induce accelerated NTN 23, a model of irreversible and progressive glomerular damage, male *PodCre* ^*+*^ */Vangl2* ^*flox/flox*^ and *Vangl2* ^*flox/flox*^ mice were pre‐immunised by subcutaneous injection of sheep immunoglobulin (0.2 mg) in complete Freund's adjuvant. This was followed by intravenous administration of sheep anti‐mouse glomerular basement membrane (GBM) nephrotoxic globulin (200 μl) 5 days later to induce nephritis. Glomerular injury follows, with capillary thrombosis and crescent formation 23.

To induce transient podocyte injury, male *PodCre* ^*+*^ */Vangl2* ^*flox/flox*^ and *Vangl2* ^*flox/flox*^ mice were injected with 10 μg/g LPS intraperitoneally 24. C57Bl/6 male wild‐type mice were also injected with either phosphate‐buffered saline (PBS) or LPS (*n* = 6 in each group) to examine glomerular levels of PCP genes.

### Histological analysis

Kidneys were fixed in 4% paraformaldehyde, dehydrated, wax‐embedded and sectioned at 5 μm. Periodic acid Schiff (PAS) staining was used to detect basement membranes and sclerosis. Glomerular morphology in 12‐week‐old male *PodCre* ^*+*^ */Vangl2* ^*flox/flox*^ and *Vangl2* ^*flox/flox*^ mice was examined by two blinded assessors and designated as normal (little PAS‐positive material and normal capillary loops) or abnormal (PAS in >50% of the tuft). At least 30 glomeruli from four separate mice in each genotype were evaluated. Results for each category were expressed as a percentage of the total glomeruli assessed. In NTN mice, thrombosis (PAS‐positive areas of occluded capillary loops) was scored using a scale of 0–4 depending on the number of quadrants affected within the glomerular tuft (each tuft divided into four quadrants for scoring purposes) 23. Fifty glomeruli were assessed per sample by a blinded assessor, and an average score was obtained for each kidney.

### Renal function assessment, immunofluorescence staining, Western blotting, electron microscopy, podocyte culture and RT‐qPCR

Details are provided in supplementary material, Supplementary materials and methods.

### Studies of human kidney tissue

We interrogated microarray data obtained from microdissected glomeruli from two independent cohorts of renal patients. Cohort I included patients with FSGS (*n* = 10), MCD (*n* = 5) and living donor (LD) healthy controls (*n* = 18) from the European Renal cDNA Bank 25 (see supplementary material, Table S1) where RNA had been hybridised to Affymetrix HG‐U133 Plus 2.0 microarrays (Santa Clara, CA, USA) 26. Cohort II included microarray data from the public domain [GEO database: http://www.ncbi.nlm.nih.gov/geo; project GSE108109; Affymetrix Human Gene 2.1 ST arrays (Santa Clara, CA, USA)]. This project includes mRNA expression data from human renal biopsies with FSGS (*n* = 16), MCD (*n* = 5) and controls (LDs) (*n* = 6). A single probe‐based analysis tool, ChipInspector (Genomatix Software GmbH, Munich, Germany), was used for transcript annotation, total intensity normalisation, significance analysis of microarrays and transcript identification based on significantly changed probes 27. The statistic algorithm in ChipInspector is a T‐test that creates artificial background data by randomly permuting the array results. Each probe has a score on the basis of its fold‐change relative to the standard deviation of repeated measurements for this probe. Probes with scores higher than a certain threshold are deemed significant. This threshold is the Delta value. The permutations of the dataset are then used to estimate the percentage of probes identified by chance at the identical Delta. Thus, a relation of significant probes to falsely discovered probes can be given for each Delta threshold. This relation is the false discovery rate (FDR), a stringency indicator. Analysis was carried out using all default settings as recommended by the software provider, with an FDR of 0% and a median false positive of 0% 27.

### Statistics

Datasets [mean ± standard error of mean (SEM)] were analysed using GraphPad Prism (GraphPad Software, La Jolla, CA, USA). Differences between two groups were analysed using an unpaired *t*‐test. When comparing more than two groups, differences were analysed using one‐way analysis of variance (ANOVA) with Bonferroni's multiple comparison *post hoc* tests. Data affected by two variables were analysed using two‐way ANOVA with Bonferroni's multiple comparison *post hoc* tests unless otherwise stated. Statistical significance was set at *p* ≤ 0.05.

## Results

### Podocyte‐specific*Vangl2* knockdown mice

**Figure 1:**
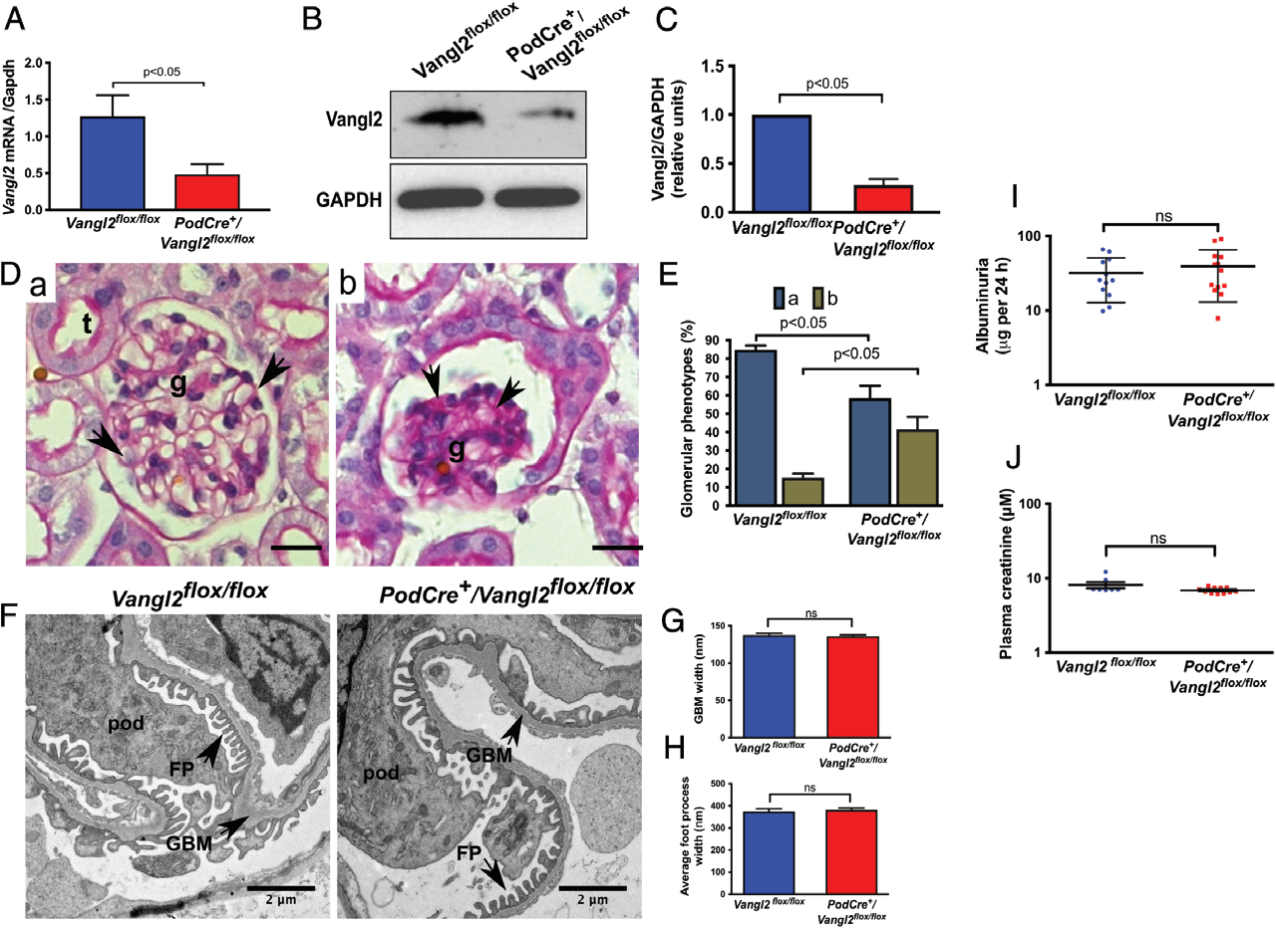
Podocyte‐specific *Vangl2* knockdown is associated with glomerular dysmorphology but no impairment of kidney function. (A) *Vangl2* mRNA expression in glomerular isolates of *Vangl2* ^*flox/flox*^ and *PodCre* ^*+*^ */Vangl2* ^*flox/flox*^ mice by RT‐qPCR (*n* = 4). (B) Representative western blot of glomerular lysates isolated from adult *PodCre* ^*+*^ */Vangl2* ^*flox/flox*^ and *Vangl2* ^*flox/flox*^ mice (*n* = 4 per genotype). (C) Semi‐quantitative densitometric analysis shows that Vangl2 protein in *PodCre* ^*+*^ */Vangl2* ^*flox/flox*^ mice is reduced (*p* < 0.05) compared with littermate *Vangl2* ^*flox/flox*^ controls. (D) Representative images of category (a) normal and (b) abnormal glomeruli used to assess glomerular morphology, scale bar = 20 μm. Normal glomeruli contain numerous patent capillary loops surrounded by a thin basement membrane stained red by PAS (arrows), whereas abnormal glomeruli have fewer patent capillary loops and increased PAS staining (arrows in b). (E) *PodCre* ^*+*^ */Vangl2* ^*flox/flox*^ mice had fewer normal (category a) glomeruli than *Vangl2* ^*flox/flox*^ controls and more category b glomeruli, (*p* < 0.05, *n* = 4 in each genotype, 30–50 glomeruli/sample). (F) Transmission electron micrographs of representative glomeruli from the two genotypes. Endo, endothelial cell; pod, podocyte. Magnification = ×10 500. Quantification of GBM width (G) and average FP width (H) (*n* = 4 in each genotype, 10 images/sample). (I) Twenty‐four‐hour albumin excretion in urine of *Vangl2* ^*flox/flox*^ (*n* = 12) and *PodCre* ^*+*^ */Vangl2* ^*flox/flox*^ mice (*n* = 12) collected at 12 weeks. (J) Plasma creatinine concentration in *Vangl2* ^*flox/flox*^ (*n =* 8) and *PodCre* ^*+*^ */Vangl2* ^*flox/flox*^ mice (*n* = 11) at 12 weeks of age. All values are presented as mean ± SEM; ns *=* not significant.

Initially, we examined the specificity of *Cre* recombination by breeding *PodCre* ^*+*^ mice with *R26R‐EYFP* mice. In the adult kidneys of *PodCre* ^*+*^ */R26R‐EYFP* mice (*n* = 2), we observed positive EYFP expression in a pattern typical of podocyte expression in the glomerular tuft (see supplementary material, Figure S1A). We subsequently bred *PodCre* ^*+*^ mice with *Vangl2* ^*flox/flox*^ mice. *PodCre* ^*+*^ */Vangl2* ^*flox/flox*^ and *Vangl2* ^*flox/flox*^ littermate controls both appeared healthy. DNA isolated from the kidney cortex of newborn (postnatal day 1) *PodCre* ^*+*^ */Vangl2* ^*flox/flox*^ mice contained both the truncated *Vangl2* allele and the intact allele (see supplementary material, Figure S1B), consistent with *Cre*‐mediated excision in podocytes that themselves represent only a proportion of *Vangl2* expressing cells in the kidney cortex. We undertook qRT‐PCR for *Vangl2* on RNA isolated from the glomeruli of 12‐week‐old *PodCre* ^*+*^ */Vangl2* ^*flox/flox*^ and *Vangl2* ^*flox/flox*^ mice using primers designed to span part of exon 4, finding that *Vangl2* transcripts containing exon 4 were reduced to 38% in *PodCre* ^*+*^ */Vangl2* ^*flox/flox*^ mice (*p* < 0.05, *n* = 4 each genotype) (Figure 1A). As assessed by Western blotting using a C‐terminal antibody, Vangl2 protein was reduced to 28% in *PodCre* ^*+*^ */Vangl2* ^*flox/flox*^ versus *Vangl2* ^*flox/flox*^ littermates (*p* < 0.05, *n* = 4 each genotype) in glomerular lysates from 12‐week‐old mice (Figure 1B, C). The remaining Vangl2 expression may be due to inefficient *Cre* recombination. Alternatively, as the mature glomerular tuft also contains endothelia and mesangial cells, Vangl2 might also be expressed in these cells. In this regard, we found *Vangl2* transcripts in cultured mouse endothelia by PCR (see supplementary material, Figure S1C). There was no significant difference in glomerular transcripts of other core PCP components (*Vangl1*, *Celsr1*, *Pk1*, *Pk2*, *Dvl1‐3*) or *Daam1* (encoding the downstream effector dishevelled associated activator of morphogenesis) (see supplementary material, Figure S2).

Next, we examined glomerular morphology and function to determine whether *Vangl2* is required for normal healthy glomeruli. Gross glomerular morphology was assessed in 12‐week‐old male *PodCre* ^*+*^ */Vangl2* ^*flox/flox*^ and *Vangl2* ^*flox/flox*^ mice using light microscopy images of kidney sections stained with PAS (Figure 1D). Significantly (*p* < 0.05) fewer normal (category a) glomeruli were observed in *PodCre* ^*+*^ */Vangl2* ^*flox/flox*^ mice (58.4 ± 6.7%) versus *Vangl2* ^*flox/flox*^ (84.8 ± 3.1%). Accordingly, the percentage of abnormal (category b) glomeruli was significantly (*p* < 0.05) higher in *PodCre* ^*+*^ */Vangl2* ^*flox/flox*^ mice (41.6 ± 6.7%) compared with *Vangl2* ^*flox/flox*^ (15.2 ± 2.2%) (Figure 1E). There was no significant difference in podocyte number between the two genotypes as assessed by quantifying the number of WT1^+^ cells in at least 30 glomeruli from each mouse (see supplementary material, Figure S3). Glomerular ultrastructure was assessed by electron microscopy (Figure 1F), and no significant difference was observed in GBM or average foot process (FP) width between *PodCre* ^*+*^ */Vangl2* ^*flox/flox*^ and *Vangl2* ^*flox/flox*^ mice (Figure 1G, H). To assess glomerular macromolecular barrier function, we quantified albuminuria over 24 h (Figure 1I), and to examine excretion of circulating small molecules, we measured plasma creatinine levels (Figure 1J). No significant differences were observed between the two genotypes for either parameter at 12 weeks.

### Genetic downregulation of *Vangl2* in podocytes worsens experimental nephritis

Thus, deletion of podocyte *Vangl2* led to modest aberrations of glomerular morphology, but this did not lead to increased albuminuria or kidney excretory failure. Therefore, we proceeded to investigate possible roles for Vangl2 in experimentally induced glomerular disease. We first used a model of irreversible and progressive glomerular damage, accelerated NTN. Here, mice are pre‐immunised with sheep immunoglobulin, and 5 days later, nephritis is induced by nephrotoxic globulin **(**see supplementary material, Figure S4A**)**. Previous work has shown that glomerular *Vangl2* transcripts increased 48 h after the initiation of NTN 8.

**Figure 2:**
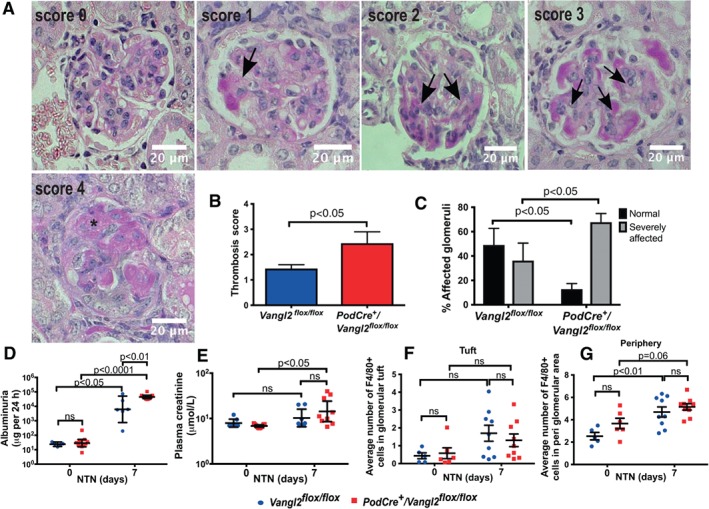
Podocyte‐specific Vangl2 knockdown exacerbates glomerular sclerosis and albuminuria following NTN. (A) Representative images of PAS‐stained glomeruli used to score thrombosis, scale bar *=* 20 μm. Arrows show areas of thrombosis (occluded capillary lumens), and * indicates a glomerular crescent. (B) Thrombosis score was higher, and a greater percentage of glomeruli were severely affected (C) (categories 2–4) in *PodCre* ^*+*^ */Vangl2* ^*flox/flox*^ mice compared with controls (*n* = 6–11 in each group, 50 glomeruli/sample). (D) Twenty‐four‐hour albumin excretion in urine and (E) plasma creatinine concentration, *n =* 6–11. Quantification of F4/80+ cells in the glomerular tuft (F) and peri‐glomerular area (G) of *Vangl2* ^*flox/flox*^ and *PodCre* ^*+*^ */Vangl2* ^*flox/flox*^ mice (*n* = 6–11 in each group, 30 glomeruli/sample). All values are presented as mean ± SEM; ns, not significant.

Seven days after disease induction, nephropathic mice displayed a range of glomerular abnormalities, including capillary thrombosis, mesangial matrix deposition, FSGS and glomerular epithelial hyperplasia, the latter representing early crescent formation (Figure 2A). We found that *PodCre* ^*+*^ */Vangl2* ^*flox/flox*^ mice had significantly increased glomerular thrombosis scores compared with *Vangl2* ^*flox/flox*^ mice (2.0 ± 0.2 versus 1.1 ± 0.3, *p* < 0.02 (Figure 2B). There was an approximately two‐fold higher prevalence of severely damaged glomeruli (scores 2–4) in *PodCre* ^*+*^ */Vangl2* ^*flox/flox*^ versus *Vangl2* ^*flox/flox*^ mice (67.7 ± 7.1% and 36.2 ± 14.3%, respectively (*p* < 0.05)) (Figure 2C). Following NTN, average 24‐h albumin excretion was significantly increased in *Vangl2* ^*flox/flox*^ mice versus levels before immunisation (*p* < 0.05, Figure 2D). Strikingly, in nephropathic mice, albuminuria was an average of 2.5‐fold higher in *PodCre* ^*+*^ */Vangl2* ^*flox/flox*^ versus *Vangl2* ^*flox/flox*^ mice (*p* < 0.01, Figure 2D). Plasma creatinine (Figure 2E) levels in the *Vangl2* ^*flox/flox*^ mice 7 days after NTN induction were similar to those before immunisation. In nephropathic *PodCre* ^*+*^ */Vangl2* ^*flox/flox*^ mice, however, creatinine significantly increased versus levels before induction of nephritis (*p* < 0.05). As creatinine is a by‐product of muscle metabolism, we also measured body weight but found no significant difference between the two nephropathic groups (see supplementary material, Figure S4B). We measured the number of WT1^+^ positive cells in at least 30 glomeruli/mouse and found that the average number of podocytes per glomerular area was not different between *PodCre* ^*+*^ */Vangl2* ^*flox/flox*^ and *Vangl2* ^*flox/flox*^ mice with NTN (see supplementary material, Figure S4C, D). There was also no difference in amounts of IgG deposited within glomeruli between nephropathic *PodCre* ^*+*^ */Vangl2* ^*flox/flox*^ and *Vangl2* ^*flox/flox*^ mice 7 days after NTN induction (see supplementary material, Figure S4E, F), indicating that the difference in disease severity between the two groups was not due to changes in glomerular antibody binding.

We also examined whether genetic deletion of podocyte *Vangl2* affected immune cell infiltration because this modulates the initiation and progression of NTN 28. We assessed numbers of F4/80^+^ positive macrophages 29 in glomerular tufts (Figure 2F) and in areas surrounding glomeruli (Figure 2G). In glomerular tufts before injury, very few F4/80^+^ positive macrophages were detected in either genotype, and there was no significant change following NTN injury. After induction of nephritis, F4/80^+^ cells around glomeruli increased in *Vangl2* ^*flox/flox*^ (*p* < 0.01) and *PodCre* ^*+*^ */Vangl2* ^*flox/flox*^ kidneys, although in the latter case, this did not reach statistical significance (*p* = 0.06), but there was no difference between genotypes.

### Deletion of *Vangl2* in podocytes alters MMP9 during NTN

**Figure 3:**
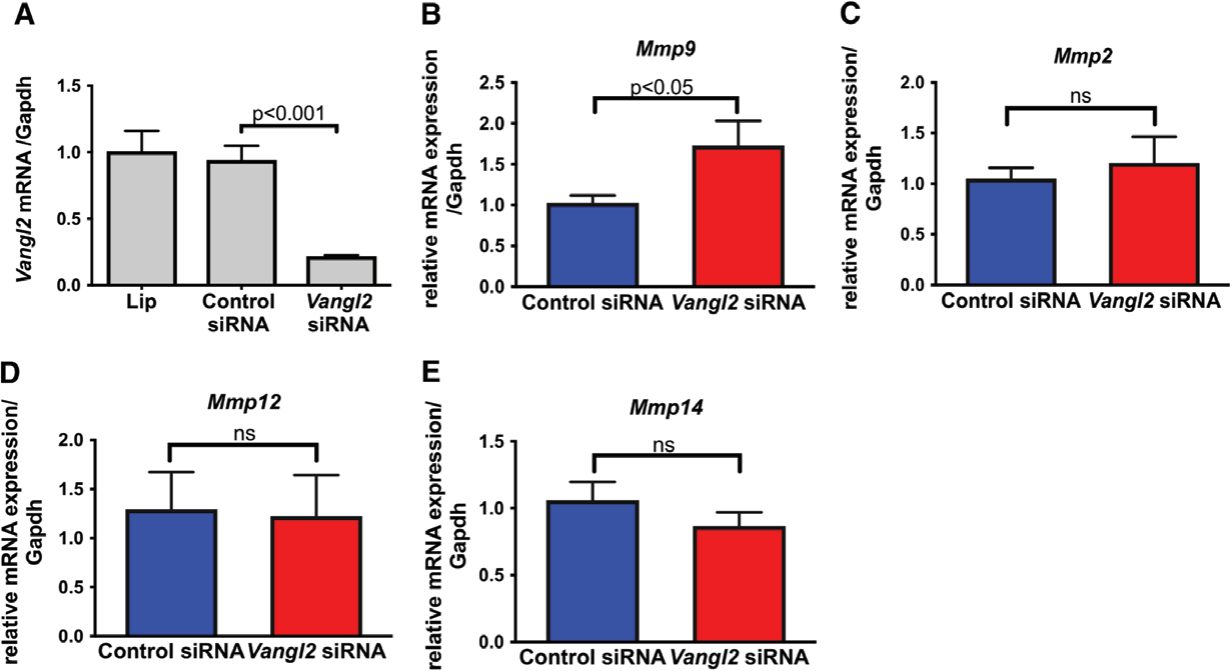
*Vangl2* siRNA knockdown increases *Mmp9* mRNA levels. Podocytes grown *in vitro* under permissive conditions were differentiated for 14 days before being transfected with control siRNA or siRNA targeting *Vangl2*. (A) Quantification of *Vangl2* mRNA levels in podocytes 48 h after transfection. Relative mRNA levels of *Mmp9* (B) *Mmp2* (C), *Mmp12* (D) and *Mmp14* (E). Experiments were repeated three to four times, and results are expressed as mean ± SEM. ns, not significant.

**Figure 4:**
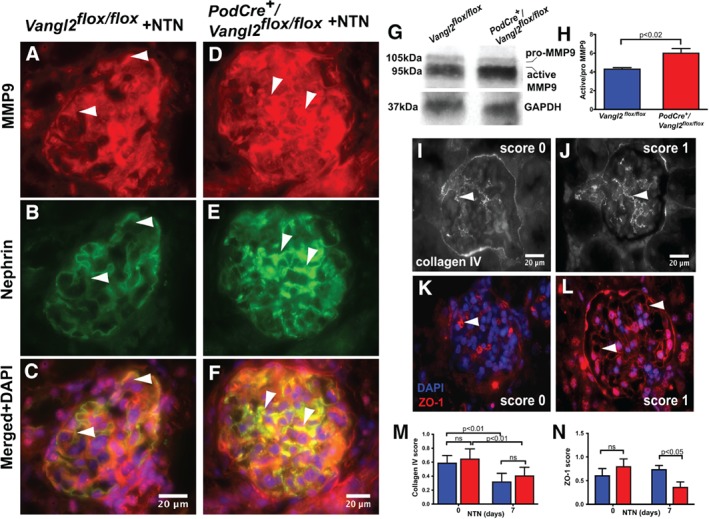
Podocyte‐specific Vangl2 knockdown increases MMP9 activity following NTN. (A–F) Representative pictures of immunostaining for MMP9 (A and D), nephrin (B and E) and merged images (C and F) in *Vangl2* ^*flox/flox*^ and *PodCre* ^*+*^ */Vangl2* ^*flox/flox*^ mice (upper and lower panel, respectively) following the administration of nephrotoxic serum. Arrowheads indicate areas of overlapping podocyte staining in both genotypes. (G) Representative western blot for MMP9 from whole kidney lysates 7 days after NTN induction. (H) The ratio of active MMP9 to pro‐MMP9 in *PodCre* ^*+*^ */Vangl2* ^*flox/flox*^ mice is increased compared with *Vangl2* ^*flox/flox*^ controls, *p* < 0.02, *n* = 6–11 in each group. (I and J) Representative images of collagen IV‐stained glomeruli scored 0 or 1. Arrowheads show positive staining in the glomerular tuft arising from the GBM surrounding the capillary loops. (K and L) Representative images of ZO‐1 stained glomeruli scored 0 or 1. Arrowheads show positive staining in the glomerulus. (M) Quantification of collagen IV and (N) ZO‐1 staining at baseline and following NTN, expressed as average score per glomerulus, in *Vangl2* ^*flox/flox*^ and *PodCre* ^*+*^ */Vangl2* ^*flox/flox*^ mice (*n* = 6–11 in each group, 30 glomeruli/sample). All values are presented as mean ± SEM; ns, not significant.

We hypothesised that genetic deletion of *Vangl2* in podocytes altered MMP activity, which could subsequently contribute to the increased disease severity of NTN observed in *PodCre* ^*+*^ */Vangl2* ^*flox/flox*^ mice. First, we knocked down *Vangl2* using siRNA in cultured mouse podocytes and measured transcript levels of *Mmp2* and *Mmp9*, both of which have been detected in podocytes 16; *Mmp12*, of which transcript levels are increased in *Vangl2* mutant lungs 14; and *Mmp14*, whose availability is elevated in zebrafish with *Vangl2* downregulation 13. *Vangl2* siRNA resulted in a >90% knockdown in *Vangl2* (Figure 3A) and significantly increased mRNA levels of *Mmp9* (*p* < 0.05) (Figure 3B) but did not affect *Mmp2*, *Mmp12* or *Mmp14* (Figure 3C–E) levels (*n* = 3 from at least three independent experiments analysed in triplicate). Next, we examined MMP9 expression in detail. MMP9 was detected by immunohistochemistry at baseline (see supplementary material, Figure S5). Following NTN, in both *Vangl2* ^*flox/flox*^ and *PodCre* ^*+*^ */Vangl2* ^*flox/flox*^ mice, MMP9 immunostaining partly spatially overlapped with nephrin, a podocyte slit diaphragm protein (Figure 4A–F). Using Western blots, we quantified MMP9 in whole kidneys of nephropathic *Vangl2* ^*flox/flox*^ and *PodCre* ^*+*^ */Vangl2* ^*flox/flox*^ mice, probing with an antibody that detects both the inactive form (105 kDa; pro‐MMP9) and the cleaved, enzymatically active form (95 kDa; active‐MMP9) (Figure 4G). The average ratio of active MMP9 to pro‐MMP9 increased by 40% in *PodCre* ^*+*^ */Vangl2* ^*flox/flox*^ versus *Vangl2* ^*flox/flox*^ tissues (Figure 4H) (6.1 ± 0.4 to 4.4 ± 0.1 respectively, *p* < 0.05, *n* = 6–11 per group).

We examined the expression of collagen IV, an MMP9 substrate 30 and a key GBM component 31, using immunofluorescent staining of kidney sections, at baseline and during NTN, with an antibody reactive to all collagen IV chains. Quantification was performed by assigning a score of 0 to glomeruli with staining in <50% of the tuft area and a score of 1 to glomeruli with staining in >50% of the tuft (Figure 4I, J). There was no significant difference between *Vangl2* ^*flox/flox*^ and *PodCre* ^*+*^ */Vangl2* ^*flox/flox*^ before induction of NTN. During NTN, the collagen IV score was reduced in both *Vangl2* ^*flox/flox*^ and *PodCre* ^*+*^ */Vangl2* ^*flox/*flox^ mice versus healthy controls (*p* < 0.01), but no difference was observed between the two genotypes (Figure 4M). We also quantified ZO‐1, a tight junction protein 32 degraded by MMP9 in cultured podocytes 33, by immunofluorescent staining using the same scoring system (Figure 4K, L). In nephropathic mice, ZO‐1 immunostaining in *PodCre* ^*+*^ */Vangl2* ^*flox/flox*^ kidneys was reduced (*p* < 0.02) to approximately half the level measured in *Vangl2* ^*flox/flox*^ organs (Figure 4N).

### Podocyte *Vangl2* deletion enhances LPS‐induced glomerular injury and modulates MMP9

**Figure 5:**
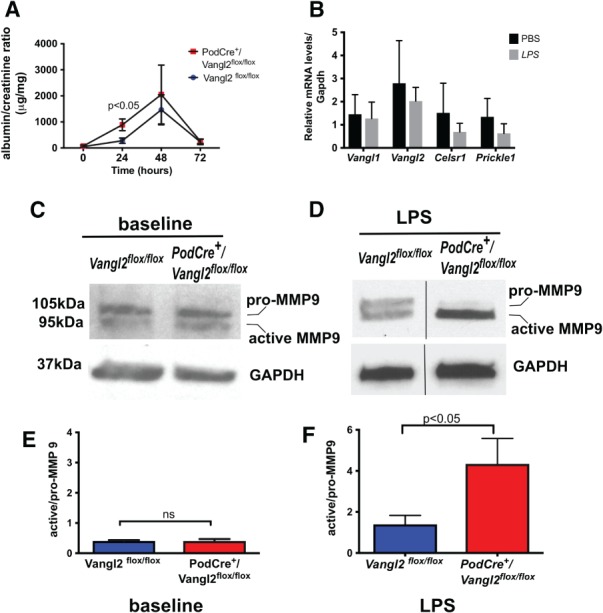
Podocyte‐specific Vangl2 knockdown exacerbates albuminuria and increases MMP9 activity following LPS injury. (A) Eight to ten‐week‐old mice were injured with LPS (10 μg/g), and albuminuria was measured at 24, 48 and 72 h. *PodCre* ^*+*^ */Vangl2* ^*flox/flox*^ mice had higher urine albumin to creatinine ratio at 24 h compared to *Vangl2* ^*flox/flox*^ controls (*n* = 5–6 per genotype), *p* < 0.05 by two‐way ANOVA and Fisher's least square difference test. (B) RT‐qPCR of core PCP genes in isolated glomeruli following injury with LPS or PBS. Representative immunoblot of MMP 9 (active and pro) in glomerular lysates from *Vangl2* ^*flox/flox*^ and *PodCre* ^*+*^ */Vangl2* ^*flox/flox*^ mice prior to (C) and (D) 24 h following LPS. Densitometric analysis using ImageJ software at baseline (E) and after LPS (F) in *PodCre* ^*+*^ */Vangl2* ^*flox/flox*^ mice compared to *Vangl2* ^*flox/flox*^ controls (*n* = 5–6 per genotype). All values are presented as mean ± SEM; ns, not significant.

Next, we determined whether Vangl2 plays a role in another glomerular disease model, LPS‐induced reversible glomerular injury. Here, podocytes are injured through the activation of the toll‐like receptor 4, leading to FP effacement within 24–48 h, followed by resolution after 72 h 24. One day after LPS administration, the urinary albumin/creatinine ratio was, on average, three‐fold greater (*p* < 0.05) in *PodCre* ^*+*^ */Vangl2* ^*flox/flox*^ versus *Vangl2* ^*flox/flox*^ mice (Figure 5A). Albuminuria continued to increase in both groups until 48 h, with a non‐significant (*p* = 0.68) tendency for higher values in *PodCre* ^*+*^ */Vangl2* ^*flox/flox*^ mice (2052 ± 1129 μg/mg) versus *Vangl2* ^*flox/flox*^ animals (1465 ±576 μg/mg). Albuminuria returned to basal levels by 72 h in both genotypes. We examined transcript levels of *Vangl1*, *Vangl2*, *Celsr1* and *Pk1* in isolated glomeruli 24 h after LPS injury and found no significant differences compared with mice injected with PBS (Figure 5B). MMP9 levels in glomerular lysates from *PodCre* ^*+*^ */Vangl2* ^*flox/flox*^ and *Vangl2* ^*flox/flox*^ were assessed using Western blotting (Figure 5C, D). In glomeruli harvested from either genotype before administration of LPS (*n* = 4–6 in each group), most MMP9 was in the inactive form (Figure 5C) with ratios of active‐MMP to pro‐MMP9 < 1 in both genotypes and no statistically significant difference between the genotypes (Figure 5E). LPS injury in *Vangl2* ^*flox/flox*^ mice resulted in an average ratio of active/pro‐MMP9 of 1.2 ± 0.4. Strikingly, the active/pro‐MMP9 ratio in *PodCre* ^*+*^ */Vangl2* ^*flox/flox*^ LPS glomeruli was 4.3 ± 1.2, significantly higher (*p* < 0.05) versus *Vangl2* ^*flox/flox*^ tissues (Figure 5F).

### Levels of PCR transcripts in human glomerular disease

To begin to examine the human relevance of this work, we assessed levels of transcripts encoded by PCP genes (*VANGL1*, *VANGL2*, *CELSR1*, *CELSR2*, *DISHEVELED 1‐3*, *FRIZZLED3*, *PRICKLE1* and *PRICKLE2*) in glomeruli from biopsies of individuals with either FSGS or MCD from two different cohorts of patients (Table 1). In cohort I, significant increases versus healthy controls were observed for all PCP transcripts examined in FSGS, including *VANGL2,* which was upregulated >1.5‐fold. Similar findings were observed in cohort II, with significant increases found in 6 of the 10 genes evaluated, one of which was *VANGL2*. In contrast, in samples from MCD patients in cohort I, only *VANGL1* and *PRICKLE1* were significantly increased versus healthy kidneys, whereas *CELSR1* levels were downregulated by 0.8‐fold. There were no significant changes in any of the PCP genes examined in the MCD patients in cohort II.

**Table 1:** Levels of PCP transcripts are altered in human glomerular disease

		Cohort I		Cohort II	
Entrez gene ID	Gene symbol	FSGS (*n* = 10) versus LDs (*n* = 18)	MCD (*n* = 5) versus LDs (*n* = 18)	FSGS (*n* = 16) versus LDs (*n* = 6)	MCD (*n* = 5) versus LDs (*n* = 6)
81839	*VANGL1*	1.37	1.24	**1.68**	ns
57216	*VANGL2*	**1.53**	ns	**1.66**	ns
9620	*CELSR1*	1.06	0.78	ns	ns
1952	*CELSR2*	1.28	ns	**2.08**	ns
1855	*DVL1*	1.25	ns	ns	ns
1856	*DVL2*	1.24	ns	**1.70**	ns
1857	*DVL3*	1.23	ns	**1.69**	ns
7976	*FZD3*	1.27	ns	ns	ns
144165	*PRICKLE1*	1.31	1.34	**1.71**	ns
166336	*PRICKLE2*	1.30	ns	0.83	ns

Single‐probe analysis for selected PCP transcripts in microdissected glomeruli from two independent cohorts of renal patients with focal and segmental glomerulosclerosis (FSGS); MCD and living kidney donors (LD) used as controls. Values are expressed as fold‐change compared to LD. Significantly upregulated genes are shown in red and downregulated genes in blue. Transcripts with a fold‐change above 1.5 or below 0.667 are displayed in bold.

## Discussion

Targeted genetic downregulation of *Vangl2* in podocytes enhanced the severity of both accelerated NTN and LPS‐induced glomerular damage. In each proteinuric model, genetic deletion of *Vangl2* in podocytes was associated with an increased ratio of active‐MMP9 to inactive‐MMP9. These observations support the conclusion that Vangl2 modulates glomerular injury in mice, at least in part by acting as a brake on MMP9, a potentially harmful endogenous enzyme. In addition, by interrogating data from two cohorts of renal patients, we report increased *VANGL2* transcript levels in glomeruli of individuals with FSGS, providing evidence that the molecule may also be involved in certain human glomerular diseases.

Previously, we 10 and others 9 showed that *Loop‐tail* (*Lp*) mice with homozygous point mutations in *Vangl2* had malformed kidneys containing fewer ureteric tree collecting duct branches and fewer mature glomeruli. However, the *Vangl2* ^*Lp/Lp*^ mouse is not an ideal model to define the specific glomerular roles of *Vangl2*. First, the mutation would affect Vangl2 in both nephron and collecting duct lineages. Accordingly, because nephrons including glomerular and collecting duct development are interdependent, the glomerular phenotype could be a secondary effect. Second, homozygous *Lp* mutants die neonatally, precluding their use in testing roles for Vangl2 in glomerular function and disease in adulthood.

To circumvent this, we used a conditional *Vangl2* ^*flox/flox*^ mouse 19 and deleted *Vangl2* specifically in glomerular podocytes. In this model, we found that there were no alterations in other PCP components in the kidney at the transcriptional level but cannot rule out possible differences in their localisation. Indeed, the *Lp* mutation affects the localisation of certain PCP components such as Pk2 34, Frizzled 3 21 and Vangl1 35. Based on our previous observations on the *Loop‐tail* mouse 10 and other evidence supporting a role for Vangl2 in podocyte morphology 36, we initially hypothesised that a lack of podocyte *Vangl2* might result in impaired glomerular morphology and function. We found that the kidneys of 12‐week‐old *PodCre* ^*+*^ */Vangl2* ^*flox/flox*^ did contain a slight but statistically significant increased proportion of morphologically abnormal glomeruli. This is likely explained by the fact that *podocin* promoter‐driven *Cre* expression, and thus *Vangl2* recombination, would start in immature glomeruli, in the capillary loop stage. Kidney function, however, appeared preserved in adults as assessed by plasma creatinine and urinary albumin levels. Our results concur with Rocque and colleagues 8, who showed that podocyte‐specific deletion of *Vangl2* using the same *PodCre* line in our study led to smaller glomeruli at 2 weeks of age, but this also did not lead to any changes in albuminuria. Furthermore, genetic deletion of podocyte *Scribble*, encoding another PCP core protein, did not lead to any changes in glomerular morphology or function 37. Collectively, these results suggest that the knockdown of an individual PCP component does not have a major effect on glomerular biology of otherwise healthy mice. On the other hand, our observations on *Vangl2* and *Celsr1* compound heterozygous mice showed a more severe foetal glomerular defect than either mouse alone 11. Future studies on mice lacking multiple PCP components could provide more insights into the potential role of this pathway in glomerular morphogenesis.

A key finding in our study is that glomerular injury, induced by nephrotoxic serum or LPS, is aggravated in mice with genetic downregulation of podocyte *Vangl2* compared with controls. Although there was no difference in albumin excretion between *PodCre* ^*+*^ */Vangl2* ^*flox/flox*^ and *Vangl2* ^*flox/flox*^ mice before glomerular injury, we cannot rule out that the increased proportion of morphologically abnormal glomeruli seen in *PodCre* ^*+*^ */Vangl2* ^*flox/flox*^ mice makes these animals more susceptible to injury. In future, inducing NTN in mice in which *Vangl2* is deleted in adulthood by an inducible *PodCre* allele 38 should help unravel whether the above modest glomerular maturation defect is playing a confounding role in worsening the severity of nephritis in mice with podocyte‐specific Vangl2 depletion.

How might Vangl2 downregulation lead to enhanced kidney injury? Possible mechanisms include cytoskeletal rearrangements affecting cell morphology 39 or changes in inflammation 14. However, in this study, we focused on the effect of Vangl2 downregulation on MMPs, which modulate tissue remodelling. First, we examined which MMPs were altered in cultured podocytes following Vangl2 downregulation and found increased transcript levels of *Mmp9*. Furthermore, in both injury models, *Vangl2* mutants had increased ratios of active‐MMP9 to inactive MMP9. MMP9 has previously been shown to be produced by podocytes 16, 33 and altered in a number of glomerular diseases, including lupus nephritis with active, fibrocellular crescents 40, DN 33, 41; viral‐associated glomerulonephritis 42, membranous 43 and hypertensive 44 nephropathy. MMP9 is also induced by activation of the toll‐like receptor 4 45, 46, which mediates the actions of LPS. The exact mechanism of how PCP proteins regulate MMPs is not fully understood. One possibility is through the regulation of vesicular trafficking 13. Alternatively, Vangl2 can regulate cell surface integrin αvβ3 expression and adhesion to fibronectin, laminin and vitronectin 47.

We subsequently examined some of the mechanisms through which increased MMP activity might aggravate glomerular disease in NTN. MMPs were originally characterised by their ability to break down ECM 48; therefore, we examined collagen IV, a key component of the glomerular ECM 31. However, we did not find any difference in collagen IV expression between *PodCre* ^*+*^ */Vangl2* ^*flox/flox*^ and *Vangl2* ^*flox/flox*^ animals with NTN. Recent studies using proteomic approaches have shown that the glomerular ECM is composed of over 140 structural and regulatory components 49, and future experiments could examine the detailed ECM proteome of nephropathic *PodCre* ^*+*^ */Vangl2* ^*flox/flox*^ and *Vangl2* ^*flox/flox*^ animals. LPS injury in mice also results in remodelling of the GBM 24 h later. A glomerular microarray study found elevated levels of transcripts encoding collagen IV α1 and α2 chains alongside laminin α5β2γ1 50, both of which normally predominate in immature glomeruli 51; we postulate that MMP9 may play a role in this process.

*In vitro*, podocyte exposure to exogenous MMP9 was shown to degrade ZO‐1, a tight junction protein 32. We also examined the distribution of ZO‐1 *in vivo* following NTN and found a significant reduction in *PodCre* ^*+*^ */Vangl2* ^*flox/flox*^ mice. Ultrastructure assessment of mice kidneys with NTN has shown that tight junction formation is an early abnormality in NTN, preceding FP effacement and podocyte bridge formation 52. The authors postulated that podocyte‐to‐podocyte tight junction function may be a compensatory mechanism to maintain glomerular filtration barrier integrity. Therefore, the loss of ZO‐1 in *PodCre* ^*+*^ */Vangl2* ^*flox/flox*^ mice with NTN may lead to filtration barrier disruption and account for the enhanced albuminuria seen in these mice. MMP9 has also been shown to upregulate podocyte integrin‐linked kinase (ILK) secretion 33, a kinase known to induce podocyte de‐differentiation and detachment in disease conditions 53; whether it is upregulated in the setting of dysfunctional PCP remains to be elucidated. Further studies inhibiting MMPs in PCP‐deficient mice would help to delineate their role in this pathway. Chemical inhibition of MMP activity has already been shown to be beneficial in some models of glomerular damage 54, 55, and based on our observations, we would predict a similar role in dysfunctional PCP‐associated glomerular damage.

To begin to examine the relevance of our mouse studies to human disease, we examined PCP transcripts in microdissected glomeruli from FSGS and MCD patients. In data from two independent cohorts of FSGS patients, significant increases versus LDs were observed for the majority of PCP transcripts examined. Importantly, in both FSGS cohorts, *VANGL2* was upregulated >1.5‐fold, suggesting this molecule may have an important biological role in FSGS. It should be noted that there were some discordant results between the two cohorts analysed (e.g. in the number of PCP transcripts found to be significantly altered), and follow‐up studies should confirm the microarray data by RT‐qPCR and assess VANGL2 at the protein level in human glomeruli. In accord with the human data, a significant upregulation of *Dvl2*, *Fz3*, *Pk1* and *Vangl2* glomerular transcripts was detected in NTN mice 48 h after the induction of disease 8. Interestingly, changes in glomerular ECM deposition are a feature of FSGS and NTN 56, 57, whereas in MCD or LPS glomerular disease, where there is no sclerosis or excess ECM deposition, the majority of PCP genes examined were unaltered. Collectively, the finding of *VANGL2* upregulation in FSGS, coupled with the observation that glomerular disease is worsened in mice deficient for *Vangl2* in podocytes, suggests that increased PCP gene expression in glomerular disease is likely to be a protective compensatory response.

## Author contributions statement

EP conceived and carried out experiments and analysed and interpreted data. EV carried out experiments and analysed data; MTL and CDC generated human mRNA data; SP, HB, KLP and MKJ carried out experiments; KEW performed electron microscopy; DJH generated and supplied the floxed Vangl2 mice; CHD was involved in study design and data interpretation; ADS generated the nephrotoxic serum, conceived experiments and was involved in data interpretation; and ASW and DAL conceived experiments and interpreted data. EP, ASW and DAL wrote the manuscript, and all authors reviewed the submitted version.

**SUPPLEMENTARY MATERIAL ONLINE**

**Supplementary materials and methods**

**Figure S1.** Characterisation of transgenic mice

**Figure S2.** *Vangl1*, *Celsr1*, *Prickle1*, *Prickle2*, *Dvl1*, *Dvl2*, *Dvl3* and *Daam1* mRNA in glomerular isolates

**Figure S3.** Immunostaining for WT‐1 and nephrin and quantification of WT1 positive podocytes

**Figure S4.** The NTN model

**Figure S5.** Immunostaining for MMP9 and nephrin in Vangl2^flox/flox^ and Pod^Cre+^/Vangl2^flox/flox^ mice at baseline

**Table S1.** Disease characteristics of patients whose samples were obtained from the European Renal cDNA Bank

## Supporting information


**Supplementary materials and methods**

**Figure S1.** Characterisation of transgenic mice
**Figure S2.** *Vangl1*, *Celsr1*, *Prickle1*, *Prickle2*, *Dvl1*, *Dvl2*, *Dvl3* and *Daam1* mRNA in glomerular isolates
**Figure S3.** Immunostaining for WT‐1 and nephrin and quantification of WT1 positive podocytes
**Figure S4.** The NTN model
**Figure S5.** Immunostaining for MMP9 and nephrin in Vangl2^flox/flox^ and Pod^Cre+^/Vangl2^flox/flox^ mice at baseline
**Table S1.** Disease characteristics of patients whose samples were obtained from the European Renal cDNA Bank
