# Supplementary material for: Vangl2, a planar cell polarity molecule, is implicated in irreversible and reversible kidney glomerular injury
Source: J Pathol. 2018 Nov 16;246(4):485–96. doi: 10.1002/path.5158 (PMC6282744; doi:10.1002/path.5158)
Supplement: Supplementary file 1 — Supplementary materials and methods [file PATH-246-485-s002.docx]

**Vangl2, a planar cell polarity molecule, is implicated in irreversible and reversible kidney glomerular injury**

**Papakrivopoulou E *et al.* J Pathol 2018 (DOI: 10.1002/path.5158)**

**Supplementary Materials and Methods**

**Citation numbers refer to the main text**

**Assessment of renal function**

Urine was collected from NTN mice by individually housing them in metabolic cages for 18 h and from LPS mice by spot collections. Albumin concentrations were measured by enzyme-linked immunosorbent assay (Bethyl Laboratories, Montgomery, TX, USA) [29]. Plasma creatinine was determined by isotope dilution electrospray mass spectrometry of venous blood [29].

**Immunofluorescence staining**

Immunofluorescence staining was performed on paraffin-embedded or frozen sections using the antibodies: CD68 (MCA497R, AbD Serotec, Oxford, UK), MMP-9 (AB19016, Millipore, Watford, UK), nephrin (GP-N2, Progen, Heidelberg, Germany), pan-Collagen IV (ab6590, Abcam, Cambridge, UK), Wilms tumor-1 (WT1; AP15857PU-S, Acris Antibodies, Herford, Germany), and zonula occludens-1 (ZO-1, 61-7300, Thermo Fisher Scientific). Species-appropriate Alexa Fluor 488 or 594 secondary antibodies were used for detection (A11073, A21207, A21210, InVitrogen) and Hoescht 33342 (Thermo Fisher Scientific) was used for nuclear staining. To measure glomerular sheep IgG deposition, frozen sections were stained with FITC-conjugated donkey anti-sheep IgG (A11016, Thermo Fisher Scientific). Images were captured for 30 glomeruli per sample and mean fluorescence intensity measured using Image J. To assess macrophages, the numbers of CD68^+^ cells were counted in at least 30 glomeruli per sample. WT1^+^ cells in at least 30 glomeruli/sample were counted to examine podocyte number; values were normalised to the glomerular area measured by ImageJ. For ZO-1 and Collagen IV staining, each glomerulus was assigned a score of 0 or 1 depending on the staining pattern in the glomerular tuft (0 for weak staining in <50% of the glomerulus; 1 for staining in >50% of the glomerulus). Thirty glomeruli were assessed per sample. For negative controls, primary antibodies were omitted.

**Western blotting**

Protein lysates were extracted from either Dynabead perfusion-isolated glomeruli [58] or whole kidneys using radioimmunoprecipitation assay buffer containing protease (cOmplete Ultra, Roche, Merck, UK) and phosphatase (PhosSTOP, Roche, Merck, UK) inhibitors. Protein (10-50 μg) was electrophoresed through SDS-PAGE gels (4-15%) and transferred to nitrocellulose membranes. Blots were probed with either mouse anti- MMP9 antibody (58803, Abcam), rabbit anti-Vangl2 antibody (OAB15535, epitope raised to the C-terminal of Vangl2, Aviva Systems Biology, California, USA) or mouse anti-glyceraldehyde 3-phosphate dehydrogenase (58803, GAPDH, Millipore) overnight followed by species-appropriate horseradish peroxidase-conjugated antibodies (P0260, Agilent Dako, Stockport, UK) and bands detected using an enhanced chemiluminescent kit. Densitometry was performed for MMP9 using Image J software. Mouse MMP9 is secreted as a latent pro-enzyme (pro-MMP; 105 kDa), cleaved into an active form (active MMP9; 98 kDa) by a variety of proteases in the extracellular space [59]. Target proteins were normalised to GAPDH and results expressed as ratios of active to pro-MMP9.

**Transmission electron microscopy**

Kidney cortex specimens from *PodCre^+^/Vangl2^flox/flox^* and *Vangl2^flox/flox^* mice (1 mm^3^) were postfixed in osmium tetroxide, dehydrated in acetone, and embedded in epoxy resin. Ultrathin sections were stained with uranyl-acetate and lead citrate and foot process and GBM width quantified using ImageJ (n=4 from each genotype, 3 glomeruli/mouse using 6-10 images/glomerulus).

**Podocyte culture**

Conditionally-immortalised mouse podocytes transgenic for a temperature sensitive SV40 large T antigen were differentiated for 14 days [10] and transfected with 10 nM siRNA specific for *Vangl2* or with a non-targeting control (both from Santa Cruz Biotechnology, Dallas, TX) using Lipofectamine RNAiMAX (Thermo Fisher Scientific) according to the manufacturer’s instructions. 48 hours after transfection, RNA was isolated and transcript levels of *Vangl2*, *Mmp2*, *Mmp9*, *Mmp12* and *Mmp14* assessed by quantitative real-time PCR.

**Quantitative real-time PCR (qRT-PCR)**

RNA was isolated from glomeruli, whole mouse kidneys or cultured podocytes. 50-500ng of RNA was used to prepare cDNA (iScript cDNA synthesis kit, Biorad, UK). qRT-PCR was performed as described [58] using *Gapdh* as a housekeeping gene. All measurements were performed in duplicate. The following primers were utilised.

| **Gene** | **Forward primer** | **Reverse primer** |
| --- | --- | --- |
| *Celsr1* | CCGCATCTTACAGCATGAGA | GCCTCGAAATGCCTCAGTAG |
| *Daam1* | GATGAACTTGACCTCACAGACAA | AGCCATGGAATTGAGCTGAT |
| *Dvl1* | GCTACTATGTCTTTGGCGACCTGTG | TGCTCTTGCTCCCTTCACTCTG |
| *Dvl2* | GGCAGTGGCACTGAGTCAGAAC | GGGGTGGAGGCATCATAACTACC |
| *Dvl3* | AGTCAGCACAGTGAAGGCAGTCG | ATCAGCATCGGGGGACCATAGAGAG |
| *Gapdh* | TGCCCCCATGTTTGTGATG | TGTGGTCATGAGCCCTTCC |
| *Pk1* | ATGGATTCTTTGGCGTTGTC | GTGCAGCATGGAAGAGTTCA |
| *Pk2* | TGGCATGCTACAGAGACCTG | CTTCCTCTGTCTTGCCCTTG |
| *Vangl1* | CACGGCAGCAGCACTACCAC | CCATCCCGTAACCCGTTTGT |
| *Vangl2* | GTGGTTCAGTTTGCCGTTTCT | GCCCGTGGAGTTATTGGT |
| *MMP9* | TCGAAGGCGACCTCAAGTG | TTCGGTGTAGCTT TGGATCCA |
| *MMP2* | ACCGTCGCCCATCATCAA | TTGC ACTGCCAACTCTTTGTCT |
| *MMP12* | TGCACTCTGCTGAAAGGAGTC | AGTTGTCCAGTTGCCCAGTT |
| *MMP14* | AGGCCAATGTTCGGAGGAAG | AGGCCAATGTTCGGAGGAAG |

**List of primers for genotyping**

| **Gene** | **Forward primer** | **Reverse primer** |
| --- | --- | --- |
| *Vangl2 floxed allele** | CCGCTGGCTTTCCTGCTGCTG | TCCTCGCCATCCCACCCTCG |
| *Δ band** | TTGACCTCAGTGCAGCGCCC | TCCTCGCCATCCCACCCTCG |
| *Podocin Cre** | GCGCTGCTGCTCCAG | CGGTTATTCAACTTGCACCA |
