# Supplementary material for: Vangl2, a planar cell polarity molecule, is implicated in irreversible and reversible kidney glomerular injury
Source: J Pathol. 2018 Nov 16;246(4):485–96. doi: 10.1002/path.5158 (PMC6282744; doi:10.1002/path.5158)
Supplement: Supplementary file 2 — Figure S1. Characterisation of transgenic mice Figure S2. Vangl1, Celsr1, Prickle1, Prickle2, Dvl1, Dvl2, Dvl3 and Daam1 mRNA in glomerular isolates Figure S3. Immunostaining for WT‐1 and nephrin and quantification of WT1 positive podocytes Figure S4. The NTN model Figure S5. Immunostaining for MMP9 and nephrin in Vangl2flox/flox and PodCre+/Vangl2flox/flox mice at baseline Table S1. Disease characteristics of patients whose samples were obtained from the European Renal cDNA Bank [file PATH-246-485-s001.pdf]

Vangl2, a planar cell polarity molecule, is implicated in irreversible and reversible kidney glomerular injury.

Papakrivopoulou E et al. J Pathol 2018 (DOI: 10.1002/path.5158)

Supplementary Figures and Tables

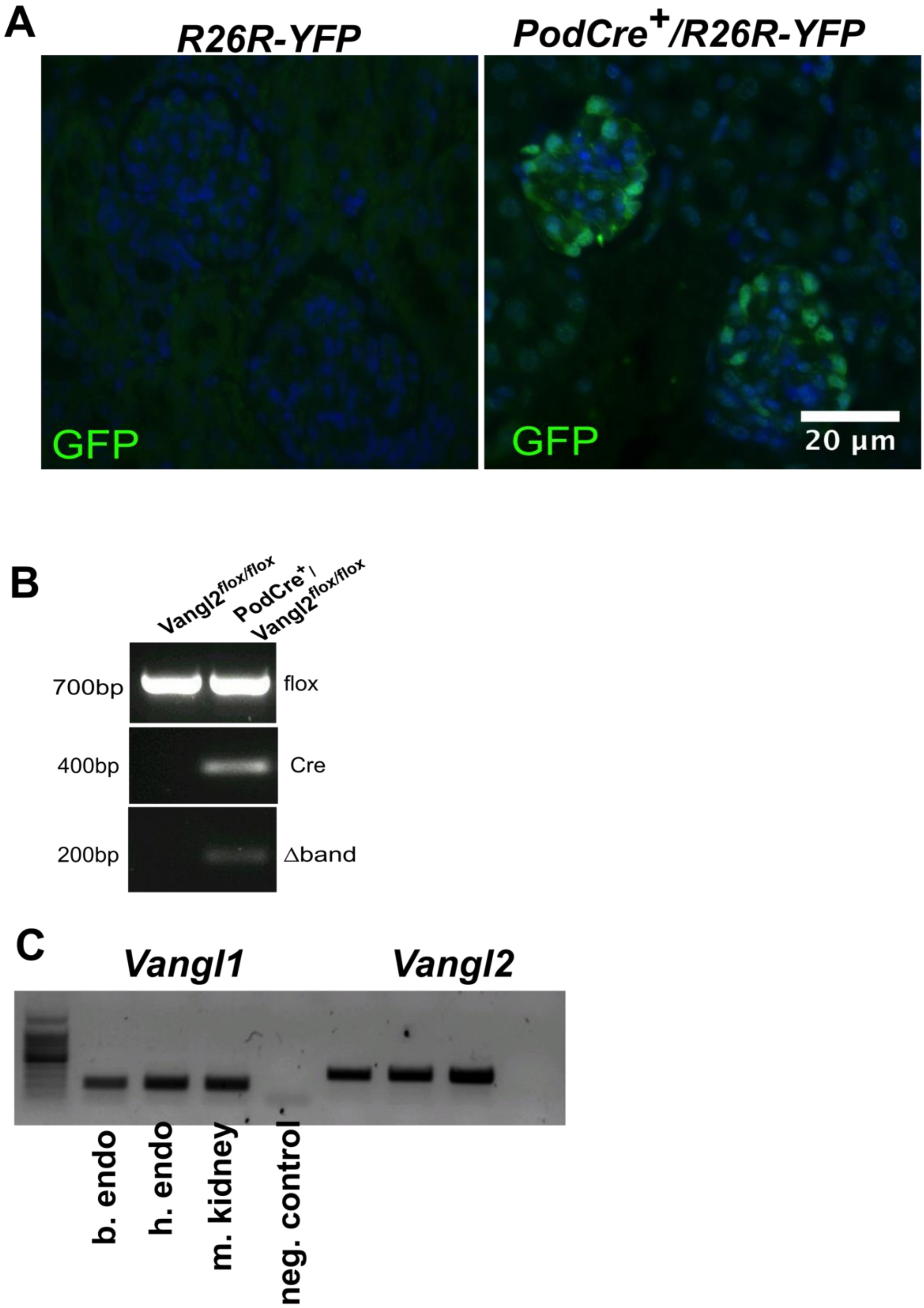

Supplementary Figure S1. (A) Representative images of immunostaining for green fluorescent protein (GFP) to detect expression of the *Podocin-Cre* promoter in *PodCre+/R26R-YFP* mice and littermate controls (*n = 2* in each group). **(B)** PCR reaction

from DNA isolated from kidney cortex of newborn mice, detecting the floxed Vangl2 allele (top panel), the *Cre* positive allele (middle panel) and the allele remaining after excision of exon 4 (bottom panel) in *PodCre*$^{+}$/*Vangl2*$^{flox/flox}$ mice. **(C)** PCR reaction from DNA isolated from endothelial cells (brain/heart) and mouse kidney detecting *Vangl1* and *Vangl2*. b.endo = brain endothelial cell; h.endo = heart endothelial cell; m.kidney = mouse kidney; neg.control = negative control.

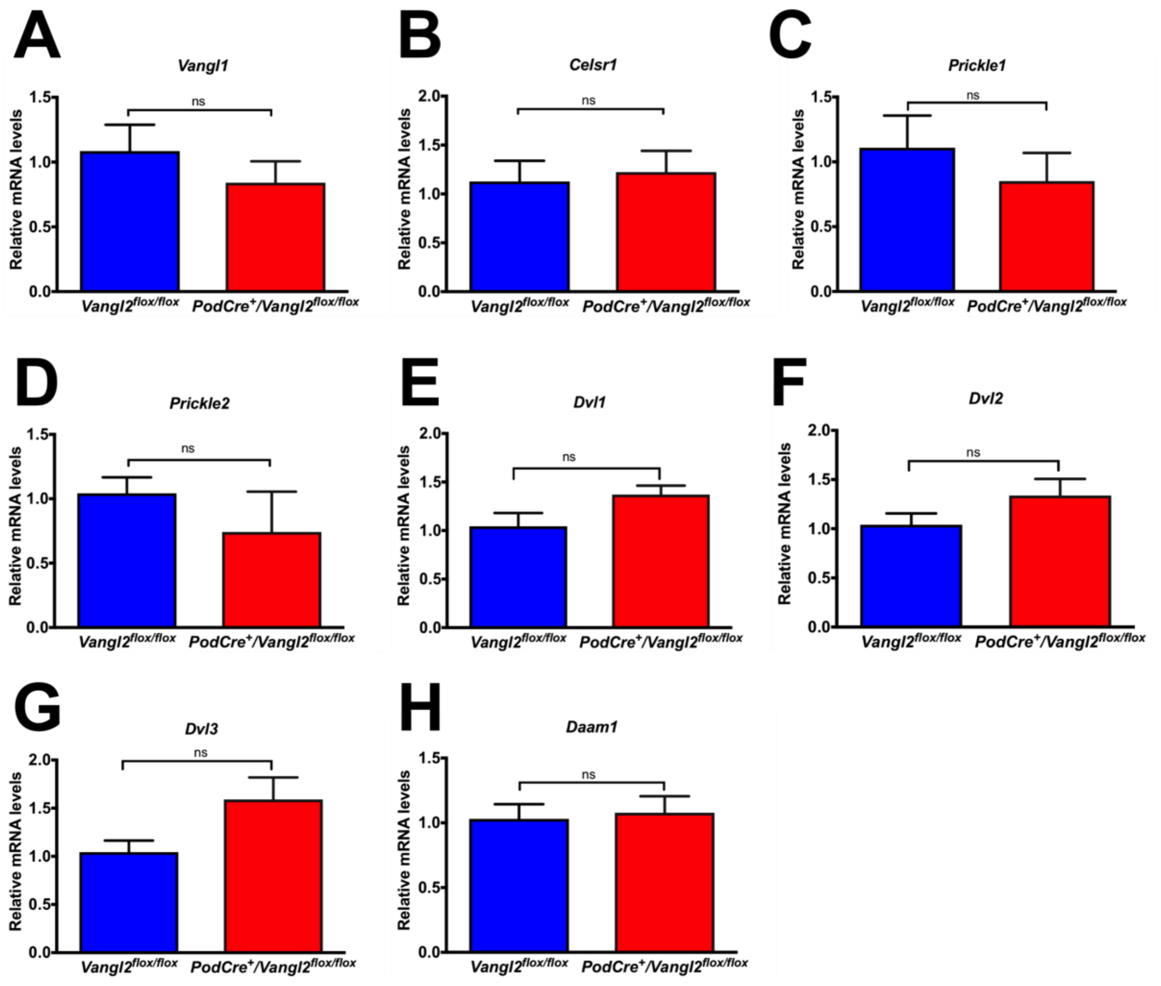

Supplementary Figure S2. Quantification of *Vangl1* (**A**), *Celsr1* (**B**), *Prickle1* (**C**), *Prickle2* (**D**), *Dvl1* (**E**), *Dvl2* (**F**), *Dvl3* (**G**), *Daam1* (**H**) mRNA in glomerular isolates of *Vangl2*$^{flox/flox}$ and *PodCre*$^{+}$/*Vangl2*$^{flox/flox}$ mice by RT-qPCR (*n* = 5). Data is presented as mean ± SEM.

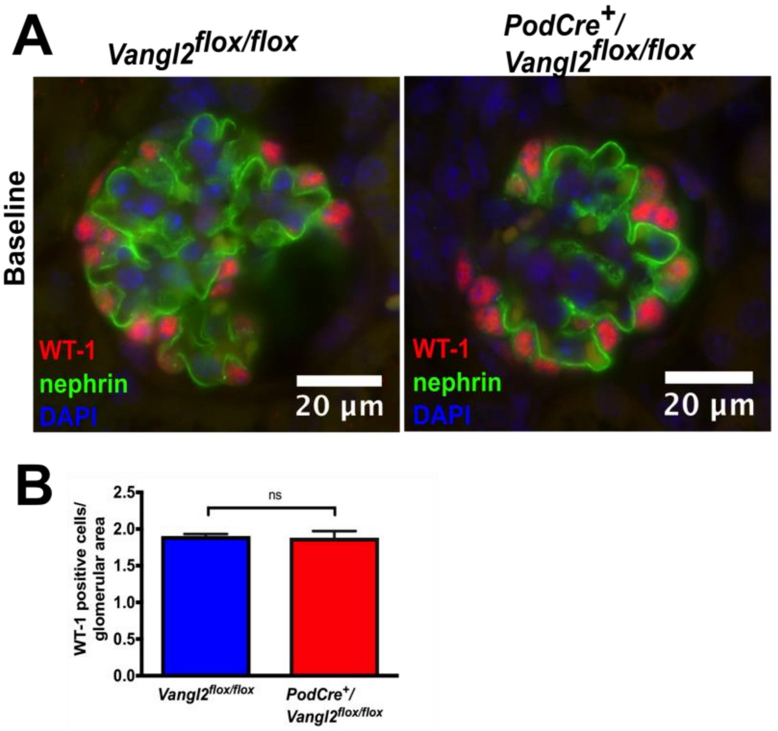

Supplementary Figure S3. (A) Representative images of immunostaining for WT-1, nephrin and DAPI in glomeruli from *Vangl2$^{flox/flox}$* and *PodCre^{+}/Vangl2$^{flox/flox}$* mice at baseline. **(B)** Quantification of the average number of WT1 positive podocytes normalised to glomerular area in *Vangl2$^{flox/flox}$* (*n* = *6*, 30 glomeruli/sample) and *PodCre^{+}/Vangl2$^{flox/flox}$* mice (*n* = *4*, 30 glomeruli/sample).

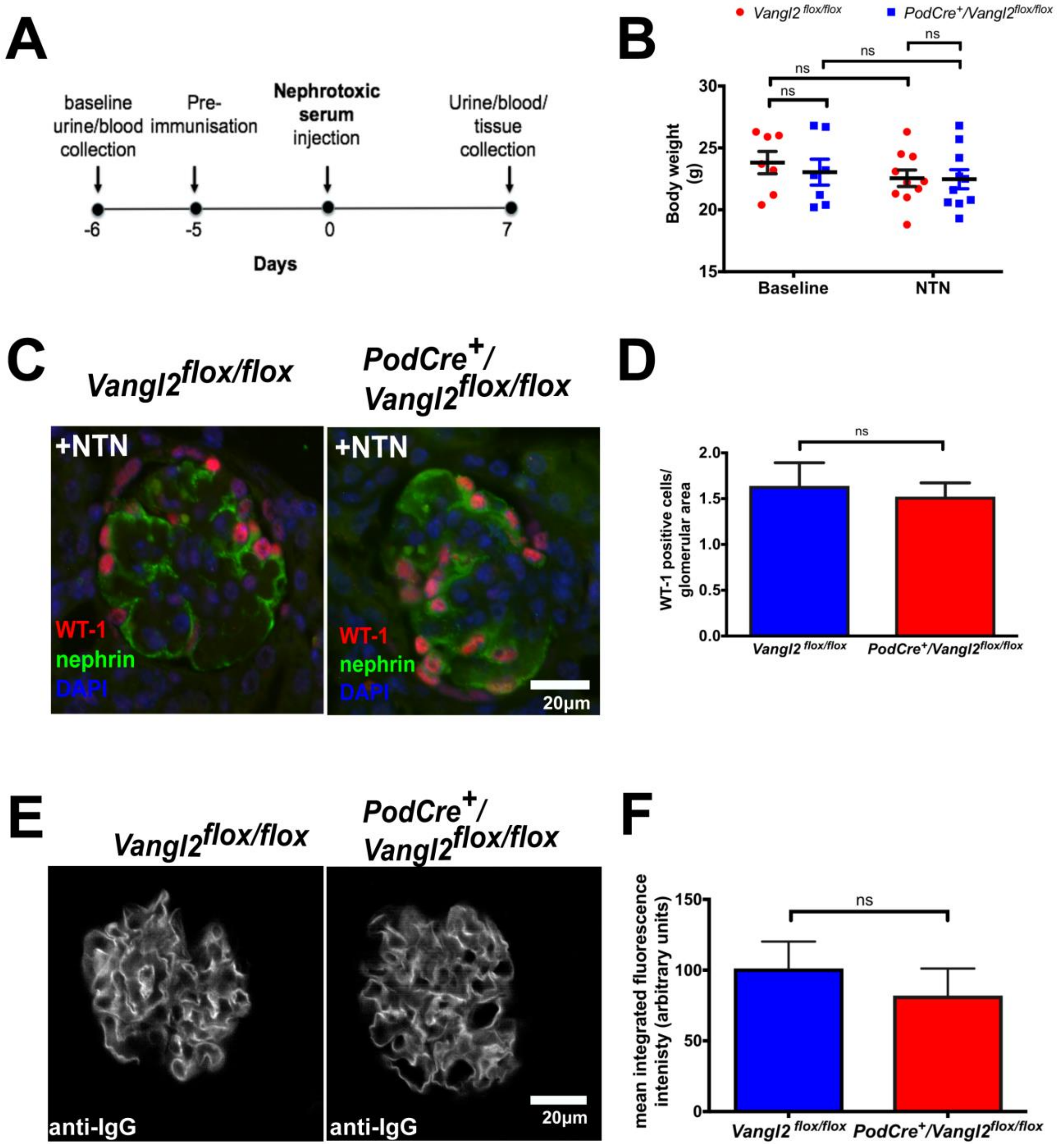

Supplementary Figure S4. (A) Time-line of NTN model. **(B)** Body weights, **(C)** representative pictures of immunostaining with an antibody against WT1 and (**D**) quantification of WT-1 positive cells/glomerular area 7 days after NTN (*n = 6-11* per genotype and 30 glomeruli/sample). (**E**) Representative pictures of immunostaining with an antibody against sheep IgG in *Vangl2*$^{flox/flox}$ and *PodCre*$^{+}$*/Vangl2*$^{flox/flox}$ mice and (**F**)

quantification of mean fluorescence in 30 glomeruli per sample (*Vangl2*$^{flox/flox}$, *n* = *6*; *PodCre*$^{+}$/*Vangl2*$^{flox/flox}$, *n* = *7*). Scale bar = 20 μm

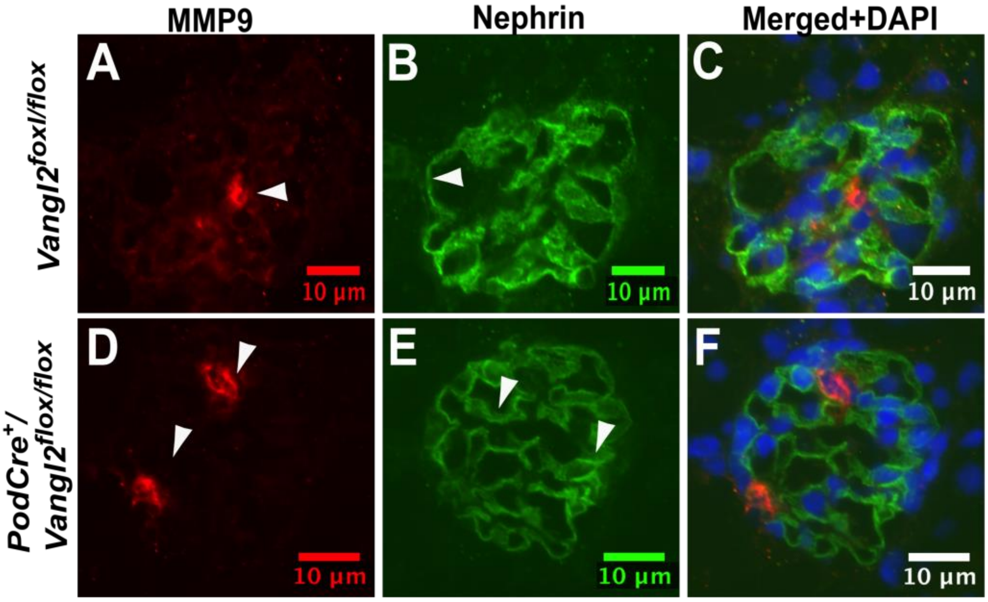

Supplementary Figure S5. Representative pictures of immunostaining for MMP9 (**A** and **D**) and nephrin (**B** and **E**) in *Vangl2*$^{flox/flox}$ and *PodCre*$^{+}$/*Vangl2*$^{flox/flox}$ mice (upper and lower panel respectively) at baseline. Arrowheads in **A** and **D** show podocyte MMP9 expression and in B and E nephrin expression. No overlap was observed in either genotype in healthy animals (**C** and **F**).

	Sex (M/F)	Age (years)	Creatinine (mg/dl)	Proteinuria (g/24h)	eGFR (MDRD) [ml/min/1.73m^3
**MCD**	**3/2**	**36.32 ±12.45 [21.13-54.37]**	**1.00 ±0.70 [0.54-2.40]**	**8.35± 6.07 [0.10-14.8]**	**109.99 ± 52.99 [22.37-165.50]**
**FSGS**	**7/3**	**43.36±12.66 [21.12-63.08]**	**1.09±0.59 [0.60-2.70]**	**3.51±2.24 [0.60-8.40]**	**81.25±33.34 [20.88-124.62]**
**LD**	**9/9**	**46.81±12.55 [22.09-62.00]**	**1.30±0.20**	*****	*****

* normal as per pre-transplant assessment

Supplementary Table S1. Disease characteristics of patients whose samples were obtained from the European Renal cDNA Bank. minimal change disease (MCD), focal segmental glomerulosclerosis (FSGS), living donor (LD), male (M), female (F), eGFR (estimated glomerular filtration rate).
